# Supplementary material for: From sequence to enzyme mechanism using multi-label machine learning
Source: BMC Bioinformatics. 2014 May 19;15:150. doi: 10.1186/1471-2105-15-150 (PMC4229970; doi:10.1186/1471-2105-15-150)
Supplement: Additional file 2 — Java code of ml2db. Additional file ml2db_code.tar.gz contains the Java source code to run the multi-label machine learning experiments and save the results to database. The code’s Javadoc is included. [file 1471-2105-15-150-S2.zip › additional file 2/ml2db/utils/doc/cern/jet/random/Pareto.html]

Pareto


JavaScript is disabled on your browser.


- Overview
- Package
- Class
- Use
- Tree
- Deprecated
- Index
- Help

- Prev Class
- Next Class

- Frames
- No Frames

- All Classes

- Summary:
- Nested |
- Field |
- Constr |
- Method

- Detail:
- Field |
- Constr |
- Method


cern.jet.random

## Class Pareto

- java.lang.Object
- - cern.colt.PersistentObject
  - - cern.jet.random.AbstractDistribution
    - - cern.jet.random.AbstractContinousDistribution
      - - cern.jet.random.Pareto

- All Implemented Interfaces:
  :   DoubleFunction, IntFunction, java.io.Serializable, java.lang.Cloneable

  Direct Known Subclasses:
  :   TruncatedPareto

  ---

    

  ```
  public class Pareto
  extends AbstractContinousDistribution
  ```

  Pareto Distribution. See the  math
  definition 
  animated definition.

  p(x) = shape\*scale^shape\*x^{-(1+shape)} for x >= scale,
  shape > 0.

  The mean is infinite if shape <= 1 and variance is infinite if
  shape <= 2.

  Instance methods operate on a user supplied uniform random number generator;
  they are unsynchronized.

  Static methods operate on a default uniform random number generator; they are synchronized.

  Version:
  :   0.1, 26-Nov-99

  Author:
  :   Andy Ogielski ato@renesys.com

  See Also:
  :   Serialized Form

- - ### Constructor Summary

    Constructors

    Constructor and Description

    `Pareto(double k,
    double alpha,
    RandomElement randomGenerator)`

    Constructs a Pareto distribution.
  - ### Method Summary

    Methods

    | Modifier and Type | Method and Description |
    | --- | --- |
    | `double` | `cdf(double x)` Returns the cumulative distribution function. |
    | `double` | `nextDouble()` Returns a random number from the distribution. |
    | `double` | `nextDouble(double k, double alpha)` Returns a random number from the distribution; bypasses the internal state. |
    | `double` | `pdf(double x)` Returns the probability distribution function. |
    | `void` | `setState(double k, double alpha)` Sets the parameters. |
    | `static double` | `staticNextDouble(double k, double alpha)` Returns a random number from the distribution with the given scale = k, and shape = alpha. |
    | `java.lang.String` | `toString()` Returns a String representation of the receiver. |

    - ### Methods inherited from class cern.jet.random.AbstractDistribution

      `apply, apply, clone, makeDefaultGenerator, nextInt`
    - ### Methods inherited from class java.lang.Object

      `equals, getClass, hashCode, notify, notifyAll, wait, wait, wait`

- - ### Constructor Detail


    - #### Pareto

      ```
      public Pareto(double k,
            double alpha,
            RandomElement randomGenerator)
      ```

      Constructs a Pareto distribution.
  - ### Method Detail


    - #### staticNextDouble

      ```
      public static double staticNextDouble(double k,
                            double alpha)
      ```

      Returns a random number from the distribution with the given scale = k,
      and shape = alpha.


    - #### cdf

      ```
      public double cdf(double x)
      ```

      Returns the cumulative distribution function.


    - #### nextDouble

      ```
      public double nextDouble()
      ```

      Returns a random number from the distribution.

      **Specified by:**
      :   `nextDouble` in class `AbstractDistribution`


    - #### nextDouble

      ```
      public double nextDouble(double k,
                      double alpha)
      ```

      Returns a random number from the distribution; bypasses the internal
      state.


    - #### pdf

      ```
      public double pdf(double x)
      ```

      Returns the probability distribution function.


    - #### setState

      ```
      public void setState(double k,
                  double alpha)
      ```

      Sets the parameters.


    - #### toString

      ```
      public java.lang.String toString()
      ```

      Returns a String representation of the receiver.

      **Overrides:**
      :   `toString` in class `java.lang.Object`


- Overview
- Package
- Class
- Use
- Tree
- Deprecated
- Index
- Help

- Prev Class
- Next Class

- Frames
- No Frames

- All Classes

- Summary:
- Nested |
- Field |
- Constr |
- Method

- Detail:
- Field |
- Constr |
- Method
